# Novel Insights into Diabetic Kidney Disease

**DOI:** 10.3390/ijms251810222

**Published:** 2024-09-23

**Authors:** Ewelina Młynarska, Dominika Buławska, Witold Czarnik, Joanna Hajdys, Gabriela Majchrowicz, Filip Prusinowski, Magdalena Stabrawa, Jacek Rysz, Beata Franczyk

**Affiliations:** 1 Department of Nephrocardiology, Medical University of Lodz, Ul. Zeromskiego 113, 90-549 Lodz, Poland; 2 Department of Nephrology, Hypertension and Family Medicine, Medical University of Lodz, Ul. Zeromskiego 113, 90-549 Lodz, Poland

**Keywords:** diabetic kidney disease, pathogenesis, treatment, molecular mechanisms, chronic kidney disease, end-stage renal disease

## Abstract

Diabetic kidney disease (DKD) is a major complication of diabetes mellitus (DM), affecting over one-third of type 1 and nearly half of type 2 diabetes patients. As the leading cause of end-stage renal disease (ESRD) globally, DKD develops through a complex interplay of chronic hyperglycemia, oxidative stress, and inflammation. Early detection is crucial, with diagnosis based on persistent albuminuria and reduced estimated glomerular filtration rate (eGFR). Treatment strategies emphasize comprehensive management, including glycemic control, blood pressure regulation, and the use of nephroprotective agents such as angiotensin-converting enzyme (ACE) inhibitors, angiotensin II receptor blockers (ARBs), sodium-glucose cotransporter-2 (SGLT2) inhibitors, and glucagon-like peptide-1 (GLP-1) receptor agonists. Ongoing research explores novel therapies targeting molecular pathways and non-coding RNAs. Preventive measures focus on rigorous control of hyperglycemia and hypertension, aiming to mitigate disease progression. Despite therapeutic advances, DKD remains a leading cause of ESRD, highlighting the need for continued research to identify new biomarkers and innovative treatments.

## 1. Introduction

DM is a disease of civilization that affects over half a billion people worldwide [1]. According to estimates by the International Diabetes Federation, this number is expected to reach 629 million by 2045 [2]. DKD is one of the most serious complications of DM, and it will occur in at least one third of patients with type I diabetes and in at least half of patients with type II diabetes [3]. Moreover, DKD is the cause of 30–47% of ESRD [4]. To diagnose DKD, the albumin to creatinine ratio in urine (ACR) >= 30 mg/g or a progressive decrease in eGFR < 60 mL/min/1.73 m^2^ must be confirmed at least twice, with an interval of three months. In order to assess the advancement of DKD, we took into account the following factors: duration of diabetes and edema disease, diabetic retinopathy, eGFR, and the amount of albumin excreted in urine. [5] Patients with diagnosed type I diabetes mellitus (T1DM) should be checked after five years from the diagnosis of diabetes in order to detect early DKD, and, in the case of patients with type II diabetes mellitus (T2DM), such tests should be performed when DM is diagnosed and every year thereafter [6]. The course of DKD begins with an increase in glomerular filtration and the periodic occurrence of microalbuminuria, the next stage is a decrease in eGFR and the constant occurrence of microalbuminuria, increasing with the duration of the disease [7]. Increased urinary protein excretion is a signal of deteriorating kidney function and a prognostic factor of disease progression and a therapeutic target [8]. DKD is the sixth most common cause of disability and the fourth cause of death in the world [9]. In this article, we put great emphasis on describing the pathogenesis of DKD, the pathways leading to its development, and the morphological changes that occur in it. We demonstrated the importance of oxidative stress, immunological disorders, lipids, and angiogenesis in pathogenesis. Additionally, we touched upon the subject of diagnostics, prevention, and treatment of diabetic kidney disease, depending on the advancement of the disease process and current complications. We presented the goals of treatment and the possible benefits based on the latest guidelines and available studies.

## 2. Pathogenesis

### 2.1. Structural Changes in Diabetic Kidneys

**Figure 1:**
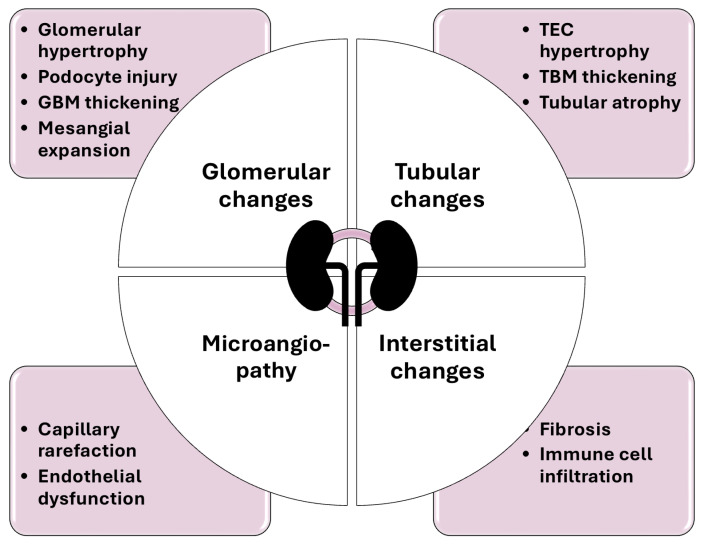
Structural changes in the diabetic kidney. GBM—glomerular basement membrane; TEC—renal tubular epithelial cells; TBM—tubular basement membrane.

Kidney injury in diabetic patients results from a combination of hemodynamic and metabolic abnormalities, which include hyperglycemia, the accumulation of advanced glycation end products (AGEs), proteinuria, and lipid overload [10,11,12,13]. Diabetic glomerulopathy is characterized by glomerular hypertrophy, glomerular basement membrane (GBM) thickening, effacement of podocyte foot processes, and the expansion of the mesangial matrix [14,15]. Glomerular endothelial cells (GECs), being a component of the filtration barrier, are exposed to damaging factors in the blood, while tubular damage is another component indispensable in DKD pathogenesis, moreover, it may occur even at the early stages of the disease [16,17,18]. Recently, research has increasingly focused on tubulointerstitial lesions in DKD, which are marked by hypertrophy and increased numbers of tubular epithelial cells, and the thickening of the tubular basement membrane (TBM) [13]. It is important to note the interplay between glomerular and tubular cell populations following cellular injury in DKD [10]. Renal fibrosis is a severe consequence of a prolonged hyperglycemic state and the consecutive activation of a cascade of multiple pathogenic mechanisms, contributing towards the loss of renal function. In particular, hyperglycemia promotes the deposition of extracellular matrix (ECM) components, such as collagen I and collagen III, underlying progressing fibrosis in DKD [19,20]. Moreover, it should be mentioned that hyperglycemia-induced microangiopathy may be both a result and subsequent trigger of further decline in DKD, and is discussed in a separate paragraph below. An illustrative overview of the structural changes affecting the kidney in DKD are provided in Figure 1.

### 2.2. Hyperfiltration

Early DKD is characterized by glomerular hyperfiltration, potentially driven by a tubular mechanism involving the upregulation of sodium-glucose cotransporters (SGLTs) and glucose transporter (GLUTs) to prevent glucosuria [11]. Persistent hyperglycemia-induced excessive filtration lowers the concentration of sodium chloride in the macula densa, which heightens sensitivity to dietary sodium chloride. This phenomenon, known as the ‘salt paradox’, correlates with a decline in glomerular filtration rate (GFR) and an increase in blood pressure (BP) after high salt intake in diabetic patients [11]. Moreover, hyperfiltration enhances oxygen consumption, thereby resulting in tubular hypoxia and tubulointerstitial fibrosis [11]. Furthermore, hyperfiltration is associated with changes in the concentrations of urine constituents. From a clinical perspective, urinary acidification dysfunction has been shown to correlate with the severity of eGFR decline, proteinuria, serum creatinine, and BUN. Moreover, renal tubular acidosis, as measured by titratable acid excretion, has been identified as an independent risk factor for the progression of DKD [17]. Thus, it represents a parameter that can have clinical utility in monitoring and managing DKD.

#### Molecular Markers of Tubulointerstitial Injury in DKD

Urinary concentrations of tubular injury markers, such as neutrophil gelatinase-associated lipocalin (NGAL) and kidney injury molecule-1 (KIM-1), are elevated in diabetic patients, even in early stages with normal albuminuria [18]. The enhanced tubular expression of NGAL and KIM-1 correlates with GFR decline as DKD progresses [16]. Non-albumin proteinuria in diabetic patients has been positively correlated with KIM-1, NGAL, and liver-type fatty acid-binding protein (L-FABP), suggesting that it may serve as an independent biomarker for tubular injury [21]. Additionally, diabetic patients with high concentrations of urine KIM-1, NGAL, cystatin-C, and angiotensinogen exhibit more rapid renal progression [22]. The elevation of urinary NGAL and KIM-1, even in the early stages of DKD, underscores their potential as early indicators of renal dysfunction, particularly in patients with normal albuminuria. Furthermore, new potential biomarkers are still under investigation. Bioinformatic analyses have specified potential immune-associated biomarkers in tubulointerstitial injury in DKD, including anterior gradient 2 (AGR2), C-C motif chemokine receptor 2 (CCR2), CCAAT enhancer binding protein delta (CEBPD), cytokine-inducible SH2-containing (CISH), C-X3-C motif chemokine receptor 1 (CX3CR1), human β-defensin-1 (DEFB1), and follistatin-like protein 1 (FSTL1), which correlate with renal function markers [23]. Given findings highlight the need for a broader focus on tubulointersitial injury, traditional markers may not fully capture the extent of renal damage in diabetes. However, while these findings are promising, further validation in clinical settings is essential to determine their practical utility in monitoring disease progression and guiding therapeutic strategies.

### 2.3. Epithelial-Mesenchymal Transition

The transformation of renal tubular epithelial cells (TECs) into cells exhibiting mesenchymal features, known as epithelial-mesenchymal transition (EMT), leads to a progressive loss of functional renal parenchyma and the development of kidney fibrosis. Targeting this process has potential clinical applications, and it has been extensively studied. These investigations aim to elucidate the underlying mechanisms of EMT and explore therapeutic strategies that could mitigate renal injury and fibrosis associated with DKD. One study investigated the phosphatase and tensin homolog (PTEN) mechanism. Increased levels of PTEN modified with a K27-linked polyubiquitin chain (PTEN K27-polyUb) have been observed in diabetic patients [24]. What is more, this finding was negatively correlated with eGFR. PTEN modification, catalyzed by mex-3 RNA binding family member C (MEX3C), alters the enzymatic activity of PTEN, converting it into a Serine/Threonine phosphatase capable of activating key regulators of the epithelial-mesenchymal transition (EMT), specifically twist basic helix-loop-helix transcription factor 1 (TWIST1), snail family transcriptional repressor 1 (SNAI1), and Yes1-associated transcriptional regulator (YAP1). Moreover, studies on both human tubular epithelial HK-2 cells and mouse tubular epithelial MCT cells revealed that this modification of PTEN can be induced by factors linked to renal fibrosis, such as transforming growth factor beta (TGF-β), connective tissue growth factor (CTGF), sonic hedgehog (SHH), interleukin (IL)-6, and hyperglycemia [24]. Another study on human HK-2 cells and diabetic rat models demonstrated that high glucose exposure reduces PTEN expression while increasing phosphoinositide 3-kinase (PI3K)/protein kinase B (Akt) signaling, which is normally suppressed by PTEN and is recognized to take part in EMT and fibrosis [25,26]. Furthermore, in the context of EMT, hyperglycemia has been shown to elevate the expression of alpha smooth muscle actin (α-SMA) while reducing the expression of E-cadherin in diabetic mice models. This shift indicates a disruption of cell–cell junctions and enhances the migratory capacity of cells—both hallmark features of EMT. Consequently, these changes lead to a compromised cellular barrier function, which can significantly impact renal integrity in the progression of DKD [24,26].

While the investigation into the mechanisms underlying EMT is essential, it is crucial to recognize the complexity of the pathways involved, as targeting this process holds promise for developing therapeutic strategies.

### 2.4. Inflammation

Chronic hyperglycemia and dyslipidemia also induce the production of reactive oxygen species (ROS), leading to oxidative stress. Oxidative damage triggers inflammatory pathways, such as mitogen-activated protein kinases (MAPK), nuclear factor kappa-light-chain-enhancer of activated B cells (NF-κB), and Janus kinase-signal transduction and transcription activation (JAK-STAT), as well as activating immune cells and promoting the release of pro-inflammatory cytokines. The persistent inflammation exacerbates kidney injury, leading to fibrosis and further loss of renal function. Together, inflammation and oxidative stress create a vicious cycle that accelerates the progression of DKD. Recruitment of immune cells relies also on vascular injury in DKD [12,13,21]. Neutrophil extracellular trapsIncreased neutrophil extracellular traps (NETs) deposition has been implicated in GEC injury, as observed in both human kidney specimens from patients with T2DM and streptozotocin-induced diabetic mouse models [14,27]. NETs exert a cytotoxic effect on GECs, inducing pyroptosis—a form of programmed cell death characterized by elevated levels of pyroptotic markers such as adipose-derived stem cell (ASC), nucleotide oligomerization domain (NOD)-like receptor protein 3(NLRP3), IL-1β, IL-18, and cleaved gasdermin D (GSDMD) [13,27,28]. Pyroptosis affects the cell membrane, leading to membrane bulging and pore formation, disrupting cellular integrity and influencing gene expression related to transmembrane transport [14].

Furthermore, NETs induce sterile inflammation via enhanced NLRP3 inflammasome activation, a phenomenon documented both in human and murine GECs [27]. What is more, the NLRP3 inflammasome pathway is significantly associated with the progression of DKD, impacting not only glomerular cells but also alsopodocytes and tubular cells [14,15,29,30,31]. Therapeutically targeting pathways such as the NLRP3/Caspase-1/GSDMD pathway [29] or adenosine monophosphate-activated protein kinase (AMPK)/mammalian target of rapamycin complex 1 (mTORC1)/NLRP3 [31] presents a promising avenue for mitigating pyroptosis, with studies conducted on rodent models supporting this potential. Notably, a caspase-1-independent NLRP3 mechanism has been observed in podocytes in studies on mice, suggesting a complex interplay of pathways involved in DKD [15].

Interestingly, in silico analyses of differential gene expression have shown that genes related to Caspase-1 and lysosomal function may serve as biomarkers for NET-related tubulointerstitial injury in DKD [32]. This highlights the importance of understanding the roles of NETs and pyroptosis in DKD pathogenesis, as they could inform future therapeutic strategies aimed at preserving renal function by modulating inflammatory and cell death pathways.

### 2.5. Lipotoxicity

Increased concentrations of urinary lysophospholipids (LPLs) have been reported in diabetic patients, a finding allied to the lipotoxicity mechanism [33]. Notably, analyses of patients with rapid progression of kidney dysfunction have identified elevated urinary lysophosphatidylcholine (LPC). Kidneys from diabetic rats were found to be highly susceptible to alterations in lipid metabolism. Further studies on lipotoxicity have revealed that exogenous LPC species or altered LPL metabolism can promote peroxisome proliferator-activated receptor-δ (PPARδ)-related genes, such as perilipin 2 (*PLIN2*), leading to the excessive intracellular deposition of lipid droplets, with the subsequent induction of organelle stress and apoptosis [34].

Furthermore, in silico analyses of immune infiltration have highlighted the involvement of macrophages M2, neutrophils, and mast cells in the pathogenesis of lipotoxicity within DKD [35]. The junctional adhesion molecule-like protein (JAML) was found to be elevated both in the serum and in the glomeruli of diabetic patients and mice, contributing to lipid accumulation in podocytes. JAML’s mechanism involves downregulating sirtuin 1 (SIRT1) and the subsequent activation of sterol regulatory element-binding protein 1 (SREBP1), a transcription factor regulating lipid metabolism [36].

Additionally, the ATP-binding cassette transporter A1 (ABCA1) is recognized for its crucial role in podocyte lipotoxicity, particularly through its involvement in cellular cholesterol efflux. ABCA1 deficiency has been shown to result in glomerular endothelial injury due to cellular cholesterol deposition, inflammation, apoptosis, and glycocalyx barrier impairment, as investigated on diabetic murine and human GECs [37,38,39].

Furthermore, coiled-coil domain containing 92 (CCDC92), a novel insulin resistance-related molecule, has been upregulated in diabetic human and murine kidney samples, especially in podocytes. What is more, molecular studies indicate that CCDC92 triggers ABCA1 degradation via PA28α-regulated proteasome activity, which ultimately contributes to podocyte lipotoxicity [39,40].

JAML, SIRT1, and ABCA1 emphasize the importance of targeting lipid dysregulation for therapeutic intervention. However, further research is needed to clarify the causal relationships among these pathways and their collective impact on DKD progression. Also, understanding how immune cell infiltration exacerbates lipotoxicity may reveal additional therapeutic targets in DKD management.

### 2.6. Organelle Dysfunction

#### 2.6.1. Lysosomal Dysfunction

Intracellular metabolic changes following the diabetic state have been reported to trigger lysosomal dysfunction, leading to disrupted autophagy. This phenomenon is particularly concerning as it arises despite the increased demand for cellular protective mechanisms due to DM-related cellular stress, secondary exacerbating kidney vulnerability, and worsening ongoing injury [41,42]. Lysosomal dysfunction can be triggered by sustained lipid overload, AGEs, hyperglycaemia, excessive ROS production, and prolonged proteinuria [41,42]. The resulting lysosomal dysfunction impairs the clearance of cellular components, which exerts a cytotoxic effect, ultimately leading to podocyte loss, tubular atrophy, and interstitial fibrosis [28,41,42].

Additionally, in line with the findings regarding lysosomal dysfunction, high glucose stimulation has been shown to suppress autophagy in mice podocyte cells, adding complexity to the underlying mechanisms involved in DKD [43].

#### 2.6.2. Mitochondrial Dysfunction

Mitochondrial dysfunction, coupled with inflammation and oxidative stress, is another component of DKD, particularly given the energy-intensive demands of podocytes and proximal tubular cells that rely heavily on mitochondrial oxidative phosphorylation and fatty acid oxidation.

Studies in diabetic mice have revealed mitochondrial abnormalities, including the excessive production of reactive oxygen species (ROS), abnormal mitophagy, and fragmentation of mitochondria [44,45,46,47]. One proposed mechanism suggests the enhanced interaction between NF-E2-related factor 2 (Nrf2) and Kelch-like ECH-associated protein (Keap1), a negative regulator of Nrf2, leading to the suppression of the Nrf2/PINK axis under hyperglycemia [45]. However, it is worth noting that the Nrf2/Keap1 interaction is known to play a broader role in oxidative stress response beyond mitochondrial health. On the other hand, research by Zhu et al. highlights the downregulation of the STING1/PINK1 pathway, in either diabetic mice or human HK-2 cells under DKD conditions [47]. Additionally, findings from T2DM mouse models suggest that activation of the mineralocorticoid receptor (MR) under high glucose ambiance disrupts mitochondrial dynamics and mitophagy through the PI3K/Akt/endothelial nitric oxide synthase (eNOS) pathway [48].

Furthermore, mitochondrial DNA damage and activation of the mitochondrial apoptotic pathway amplify inflammation through NFκB and STAT3, leading to tubular injury and apoptosis [44,45]. Moreover, the reduction of the peroxisome proliferator-activated receptor γ coactivator-1α (PGC-1α), a key regulator of mitochondrial dynamics and bioenergetics in diabetic mice, correlated with reduced fatty acid β-oxidation and subsequent intracellular lipid accumulation [46]. Disruptions in lipid metabolism, including the formation of lipid droplets and the accumulation of sphingosine-1-phosphate (S1P), may also result from the hyperglycemia-related overexpression of cytosolic phospholipase A2 (cPLA2) [49].

Moreover, the decline in sirtuin 3 (SIRT3), a critical mitochondrial deacetylase playing a vital role in maintaining mitochondrial function by regulating fatty acid oxidation and protecting against oxidative stress in diabetic mice kidneys, exacerbates oxidative stress by increasing the acetylation of superoxide dismutase 2 (SOD2) and isocitrate dehydrogenase 2 (IDH2), further impairing mitochondrial function [44]. Notably, mitochondrial dysfunction and inflammation are closely intertwined. Upregulation of interferon-stimulated genes and the activation of the cyclic GMP-AMP synthase-stimulator of interferon genes (cGAS-STING) pathway, responsible for detecting cytosolic DNA and initiating an inflammatory response, reflects an enhanced innate immune response. This immune activation in diabetic murine kidneys is corroborated by elevated levels of pro-inflammatory cytokines and markers, including monocyte chemoattractant protein 1 (MCP-1), tumor necrosis factor (TNF), IL-6, and the tissue inhibitor of metalloproteinase 1 (TIMP-1) [44].

### 2.7. Vascular Dysfunction

One of the key elements of DKD is microangiopathy, marked by aberrant angiogenesis in the early stages and glomerular capillary rarefaction in advanced stages. This vascular dysfunction is closely tied to GBM thickening, glomerular hypertrophy, and mesangial expansion, which collectively exacerbate renal damage [20]. The altered resistance of peri-glomerular arteries due to increased levels of vasoactive factors in DM further complicates glomerular hemodynamics [11]. 

A central factor in these vascular changes is the dysregulated expression of vascular endothelial growth factor (VEGF)-A. While VEGF-A is crucial for maintaining the integrity of glomerular capillaries, and is highly expressed in podocytes and tubular cells, its upregulation in a hyperglycemic state drives excessive angiogenesis, which may initially appear protective but ultimately becomes pathological. The activation of the VEGF-A/VEGFR2 signaling pathway is implicated in excessive angiogenesis in DKD, with downstream pathways including phospholipase C (PLC)-γ/protein kinase C (PKC), PI3K, and p38-MAPK, promoting endothelial cell proliferation, migration, survival, and increased vascular permeability [20]. This imbalance between pro-angiogenic signals and the structural needs of the kidney contributes to the progressive capillary damage seen in DKD

Nitric oxide (NO) and eNOS are also critical regulators of glomerular function, with their dysregulation having significant consequences for DKD progression. Studies have shown that renal expression and activity of eNOS are increased in the early stage of DM, potentially contributing to vasodilation and hyperfiltration, whereas the progression of DM eNOS levels decreases, leading to reduced NO bioavailability and promoting the further advancement of the disease [50]. The reduction in NO disrupts the VEGF-A/NO axis, worsening endothelial dysfunction and promoting renal fibrosis, inflammation, and thrombosis [20,50]. What is more, the decline in NO production, coupled with reduced VEGF-A signaling, creates a vicious cycle of endothelial dysfunction, neoangiogenesis, and worsening kidney function [20,50]. The dynamic nature of eNOS and NO regulation highlights a critical area where early interventions could have a protective effect, but by the later stages, the deficiency of NO may accelerate disease progression.

Furthermore, the influence of genetic polymorphism in eNOS further complicates the risk profile for DKD. Variations in the eNOS gene may predispose certain individuals to more severe forms of DKD, suggesting a need for personalized medical approaches that consider genetic predisposition [51].

The imbalance in angiopoietin signaling, particularly the shift in the angiopoietin 1 (Ang1)/angiopoietin 2 (Ang2) ratio, plays a pivotal role in capillary destabilization and heightened vascular permeability in DKD. Ang1 is essential for maintaining the stability and confluence of GECs, and the stability of their interaction with pericytes, but its expression is suppressed under diabetic conditions. In contrast, the dominance of Ang2 leads to a loss of pericytes due to damage to cell junctions and the basal membrane, resulting in pericyte loss and further exacerbating microvascular damage [20]. This dysregulation highlights a significant vulnerability in the glomerular microvasculature, where the loss of Ang1’s stabilizing influence accelerates capillary rarefaction and vascular dysfunction.

Additional studies have shown dysregulation of another angiogenic factor in DKD, including vasoinhibins (VASHs) [20]. This points to a complex network of signals governing microvascular health, which may be affected by hyperglycemia and chronic inflammation. Several proangiogenic factors have been identified as being upregulated in DKD, such as leucine-rich α-2-glycoprotein 1 (LRG1), which enhances TGF-β/activin receptor-like kinase 1 (ALK1)-Smad1/5/8 signaling in GECs, promoting glomerular angiogenesis [20,52]. Interestingly, studies in diabetic mice demonstrated that LRG1-induced glomerular hypertrophy precedes the onset of overt DKD and even the rise in VEGF expression, positioning LRG1 as an early mediator in the development of DKD [53]. This suggests that LRG1 could be a valuable biomarker for the early detection of DKD or a target for early intervention before significant kidney damage occurs.

Another molecule involved in early diabetes-induced angiogenesis is the adhesion G protein-coupled receptor-56 (GPR56). GPR56 is upregulated in diabetic mouse kidneys, and its activation triggers GEC injury by reducing the expression of eNOS, driven by activation of the Gα12/13-RhoA pathway and subsequent Gαi-mediated inhibition of the cAMP/PKA pathway [54,55]. The reduction of eNOS activity further diminishes NO bioavailability, compounding endothelial dysfunction and promoting vascular instability. This interplay between GPR56 signaling and endothelial dysfunction underscores how multiple signaling pathways converge to disrupt glomerular vascular homeostasis in DKD.

### 2.8. Epigenetic Changes

**Figure 2:**
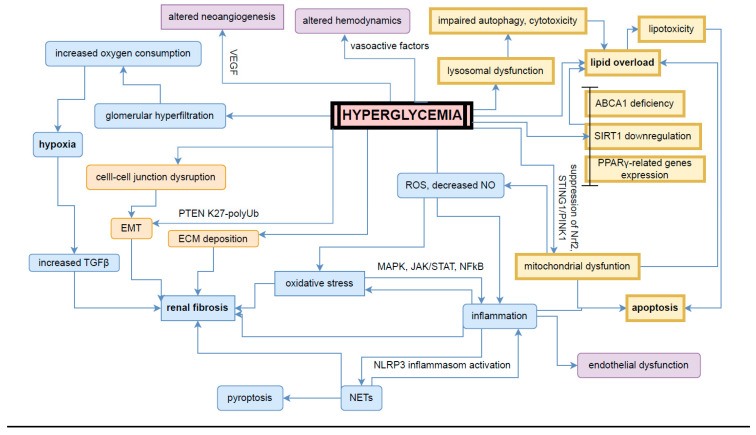
Mechanisms of hyperglycemia in diabetic kidney disease. VEGF—vascular endothelial growth factor; PTEN K27-polyUb—phosphatase and tensin homolog (PTEN) K27-polyubiquitinated; TGF-β—transforming growth factor β; NLRP3—NLR family pyrin domain containing 3; MAPK—mitogen-activated protein kinases; JAK/STAT—Janus kinase/signal transducers and activators of transcription; NFκB—nuclear factor kappa-light-chain-enhancer of activated B cells; NETs—neutrophil extracellular traps; EMT—epithelial–mesenchymal transition; ROS—reactive oxygen species; NO—nitric oxide; ECM—extracellular matrix; STING/PINK1—stimulator of interferon genes/PTEN-induced kinase 1; ABCA1—ATP-binding cassette sub-family A member 1; SIRT1—sirtuin 1; PPARγ—peroxisome proliferators-activated receptor γ; Nrf2—nuclear factor erythroid 2-related factor 2.

In addition to the aforementioned mechanisms underlying DKD progression, such as those diagrammed in Figure 2, ongoing research emphasizes the importance of epigenetic changes in regulating gene expression related to DKD pathogenesis. These epigenetic modifications, including DNA methylation, histone modification, non-coding RNA, and microRNA, may arise from hyperglycemic conditions or act independently as contributors to DKD development. Their role is increasingly recognized as a key layer of regulation that adds complexity to the disease’s progression [56,57].

One crucial concept related to these epigenetic alterations is “metabolic memory”, which refers to the long-term detrimental effects of hyperglycemia even after glycemic control has been restored. Epigenetic changes, together with the accumulation of AGEs, are considered fundamental drivers of metabolic memory. This phenomenon complicates the course of DKD and poses significant challenges for treatment, as the lasting impact of hyperglycemia persists despite subsequent interventions [56,57]. Consequently, addressing metabolic memory through epigenetic-targeted therapies could be a promising avenue for improving long-term outcomes in diabetic patients, potentially mitigating the progression of DKD, even after hyperglycemia has been managed.

### 2.9. Overview of the Pathogenesis in DKD

Understanding individual variability in response to hyperglycemia and the consequent kidney damage is essential for developing personalized therapeutic strategies in DKD. Utilizing biomarkers such as NGAL and KIM-1 can facilitate tailored treatment plans, thereby improving outcomes for patients with varying disease severities.

Moreover, the concept of “metabolic memory” and the dynamic nature of eNOS and NO regulation underscore the importance of early implementation of glucose-lowering agents and rigorous blood glucose control, which are crucial for both the management and prevention of DKD.

Furthermore, emerging anti-inflammatory therapies that target the NLRP3 inflammasome or organelle dysfunction represent promising avenues for mitigating inflammation and fibrosis, two key contributors to the progression of DKD.

The involvement of mitochondrial DNA damage and subsequent NF-κB-mediated inflammation is another area where more critical evaluation is needed. While this axis clearly contributes to tubular injury and apoptosis, it is unclear whether targeting these inflammatory pathways will sufficiently restore mitochondrial function, or if mitochondrial-targeted therapies need to be complemented by anti-inflammatory strategies.

Given the complexity of this disease, combination therapies that simultaneously target multiple pathways may enhance treatment efficacy while minimizing side effects. Such an integrative approach holds significant potential for improving patient outcomes and advancing the management of DKD, and is discussed further in the sections below.

## 3. Diagnosis of Diabetic Nephropathy

DKD is characterized by the deterioration of kidney function, a decrease in the GFR, the presence of diabetic retinopathy, and proteinuria. The primary diagnostic indicators of DKD include persistent albuminuria (category A3, severely increased), which coexists with diabetic retinopathy and is observed in the absence of other kidney diseases [58]. The most important and commonly used diagnostic marker for DKD is albuminuria, which can be measured using the ACR on spot urine samples (the samples should ideally be collected early in the morning) or a 24-h urine collection. Kidney function should be assessed using serum creatinine-based eGFR calculations, preferably employing the chronic kidney disease epidemiology collaboration (CKD-EPI) formula due to its superior performance in the eGFR range of 60–90 mL/min/1.73 m^2^ [59]. The 2012 Kidney Disease: Improving Global Outcomes (KDIGO) guidelines for chronic kidney disease (CKD) recommend classifying albuminuria into three categories based on the concentration of albumin in the urine, as outlined in Table 1 [60].

According to the American Diabetic Association (ADA) recommendations, screening for DKD should be initiated at the time of diagnosis in patients with T2DM and should be repeated annually thereafter [61]. Data indicate that at the time of T2DM diagnosis, albuminuria is present in 7% of patients [62]. In patients with T1DM, albuminuria testing is recommended five years after the diagnosis and annually thereafter. The development of DKD is rare within the first 10 years following a diagnosis of T1DM, however, between 10 and 20 years post-diagnosis, the incidence of DKD increases by approximately 3% per year [59]. After 20 years, the incidence rate decreases, meaning that individuals with normal kidney function and normal urinary albumin excretion after 30 years of living with T1DM are at a reduced risk of developing DKD [63]. DKD is a prevalent complication among patients with DM, with severe albuminuria (A3) occurring in about 15% of cases, and another 15% of patients exhibiting moderate albuminuria (A2) [64].

If an abnormal increase in albuminuria is detected, it should be confirmed with repeat testing over three to six months. If at least two out of three ACR tests show abnormal urinary albumin levels, increased albuminuria can be confirmed in the individual [61]. These patients should undergo a comprehensive assessment for the presence of comorbidities, particularly retinopathy and other diabetes-related complications.

Kidney biopsy is rarely performed in the diagnostic process for diabetic nephropathy (DN), however, the characteristic histological changes associated with DKD are described in the international classification system. There are four classes of histological change typical of DN. In Class I, mild or non-specific light microscopy (LM) changes occur, accompanied by electron microscopy (EM)-proven GBM thickening. Class IIa involves mild mesangial expansion, while Class IIb is marked by severe mesangial expansion. Class III is distinguished by the presence of nodular sclerosis, also known as Kimmelstiel–Wilson lesions. In the final stage, Class IV, advanced diabetic glomerulosclerosis is present [65].

## 4. Management of Diabetic Kidney Disease

Since multimorbidity is common in patients with DM and CKD, it is recommended that they should be treated with comprehensive care and a holistic approach to reduce their risks of cardiovascular disease (CVD) and kidney disease progression [66]. Lifestyle modification should be the foundation of this approach and should include optimized diet and exercise, smoking cessation, and weight management [67]. Other interventions should be layered upon that foundation: first-line drug therapy, additional drugs for heart and kidney protection, as well as additional interventions to further control risk factors such as glycemic and BP control, and lipid management [67]. Risk factors in these patients should be reassessed regularly every three to six months [67].

### 4.1. Glycemic Control

Strict glycemic control is crucial for preventing the development and progression of DKD [68]. Studies such as the UKPDS [69] and the ADVANCE [70] have shown that glucose-lowering therapies reduced the risk of diabetic microvascular complications, including DKD.

Both the KDIGO and the ADA recommend using hemoglobin A1c (HbA1c) to monitor long-term glycemic control [66]. It is recommended to assess HbA1c levels twice per year in patients with stable DM and as often as quarterly in patients who do not reach their treatment goals or after a change in their therapy [66]. However, as observed in some studies, the accuracy of HbA1c measurement declines in advanced stages of CKD, therefore, in such cases, data derived from continuous glucose monitoring (CGM) can be used to assess glycemia [67,71,72].

A study by Currie [73] et al. showed that, among patients with DM and CKD, both high and low mean HbA1c values were associated with an increased all-cause mortality and cardiac events. These findings suggest that a target HbA1c value should be individually optimized based on the patient’s needs [67]. The KDIGO recommends an individualized HbA1c target of <6.5% to <8.0% in patients with diabetes and CKD who are not treated with dialysis [67]. Factors deciding the individual HbA1c target include the severity of CKD, macrovascular complications, comorbidities, life expectancy, hypoglycemia awareness, resources for hypoglycemia management, and propensity of treatment to cause hypoglycemia [67]. The ADA recommends an individual HbA1c goal, starting with <7% in most patients [66,74].

The ADA 2022 Standards Care and the KDIGO 2022 guidelines both recommend metformin and an SGLT2 inhibitor (SGLT2i) as first-line drug therapy for patients with CKD and diabetes [66]. In patients with an eGFR ≥30 mL/min per 1.73 m^2^, such a combination can be used safely and effectively [67]. SGLT2i are recommended as a part of comprehensive therapy since they not only lower blood glucose but have also been proven to reduce the risk of CKD progression and CVD complications in several studies [67,75,76,77]. Therefore, they should be added to therapy even in patients who reach their glycemic goals [67]. Treatment with SGLT2i can be started with the eGFR rate of 20–29 mL/min/1.73 m^2^ and continued at an even lower eGFR if previously initiated and well tolerated. Yet, SGLT2i have a weak-to-no effect on glycemia at lower rates of eGFR, and are used mainly for other benefits [66,67]. Metformin is effective at lowering blood glucose and HbA1c levels, and has a lower risk for hypoglycemia compared to other glucose-lowering drugs [67,78,79]. Metformin has also been proven to be effective in preventing weight gain [78]. The dose of metformin should be adjusted if the eGFR is <45 mL/min per 1.73 m^2^, and the treatment should be stopped if the eGFR declines to <30 mL/min per 1.73 m^2^, or when the patient starts dialysis treatment [67]. If the patient cannot tolerate metformin, therapy with SGLT2i alone may be reasonable [66].

If the glycemic targets are not reached with base therapy or the patients are unable to take these drugs, additional glucose-lowering drugs can be added [66,67]. Both the KDIGO and the ADA recommend using long-acting GLP-1 receptor agonists as the preferred additional drug [66]. GLP-1 receptor agonists have been shown to improve glycemic control and promote weight loss [67]. Moreover, they have been observed to reduce the incidence of major adverse cardiovascular events (MACE) in diabetic patients with HbA1c > 7.0% and a high cardiovascular risk [80,81,82,83]. Meta-analyses of randomized trials by Sattar et al. [84] have shown that GLP-1 receptor agonists reduced the risk of broad composite kidney outcome, which included factors such as the development of new severely increased albuminuria, a decline in eGFR, a rise in serum creatinine, a progression to kidney failure, or death from kidney disease, as well as the risk of all-cause mortality in T2DM patients. The KDIGO recommends that GLP-1 receptor agonists may be a preferred drug for patients with DM, CKD, and obesity to promote weight loss [67]. The preferred GLP-1 receptor agonists, which are known to possess CVD and CKD benefits, include liraglutide, semaglutide, albiglutide, and dulaglutide [66]. GLP-1 receptor agonists can be used safely and effectively with eGFR as low as 15 mL/min/1.73 m^2^ and in dialysis patients [66,67]. However, their beneficial effect on cardiovascular risk is smaller among individuals with eGFR < 60 mL/min/1.73 m^2^ [84].

Glycemic control may be challenging in patients suffering from an advanced CKD, with eGFR < 30 mL/min/1.73 m^2^, or receiving dialysis, because many medications are restricted and data from trials concerning treatment strategies are lacking [66]. For patients with T1DM, insulin is the only approved therapy, and doses of insulin may need to be adjusted with the progression of CKD [66,85]. In patients with T2DM, an advanced CKD is a risk factor for hypoglycemia, therefore antidiabetic drugs that do not increase the risk of hypoglycemia are preferred [66,86,87]. Some of the dipeptidyl peptidase 4 inhibitors can be used with eGFR <30 mL/min/1.73 m^2^ and in dialysis, therefore, they are a safe option for patients who cannot be treated with GLP-1 receptor agonists [66]. Insulin is often needed when other medications are contraindicated, not tolerated, or insufficient [66]. For T2DM patients after a kidney transplant, metformin seems to be a reasonable choice of medication [66]. SGLT2i appear to be promising, however, there are not enough data to support any recommendation [66,88,89].

### 4.2. Lipid Management

Dyslipidemia is a common comorbidity in patients diagnosed with DKD [90]. High levels of total cholesterol, low-density cholesterol, and triglycerides are also a risk factor for atherosclerotic CVD and the development and progression of DKD [91].

The 2013 KDIGO Clinical Practice Guideline for Lipid Management in Chronic Kidney Disease recommends statin treatment in most adult patients with DM and CKD who are not treated with dialysis [92]. These patients include adults older than 50 years old with CKD and eGFR ≥ 60 mL/min/1.73 m^2^, and adults between the ages of 18 and 49 with CKD and DM, known coronary heart disease, prior ischemic stroke, or an estimated 10-year incidence of coronary heart disease death or non-fatal myocardial infarction >10% [92].

The ADA recommends a moderate-intensity statin therapy as a form of primary CVD prevention for all adults with DM between the ages of 40 and 75, for adults between the ages of 20 and 39 with additional atherosclerotic CVD risk factors, and for adults older than 75 years old, with an individual decision on treatment initiation [66]. These recommendations may exclude patients treated with hemodialysis, as the primary prevention of atherosclerotic CVD events with a statin has been proven to be ineffective [93,94,95]. For secondary prevention, the ADA recommends high-intensity statin therapy [66]. If the intensification of statin treatment is needed, an addition of ezetimibe or a PCSK-9 inhibitor is recommended based on CVD risk and achieved low-density cholesterol levels [66].

The Association of British Clinical Diabetologists (ABCD) and the UK Kidney Association (UKKA) in their guidelines from 2024 recommend the initiation of statin therapy with 20 mg atorvastatin at all stages of DKD [96]. For patients in stages G1–G3a of kidney disease who do not reach their treatment targets, it is recommended to consider a higher dose or combination therapy with ezetimibe [96]. In cases of statin intolerance in stages G1–G3a of kidney disease, ezetimibe therapy alone or ezetimibe in combination with bempedoic acid if treatment goals are not reached with ezetimibe alone, is recommended [96].

### 4.3. BP Management

Hypertension is commonly diagnosed in patients with DKD [97]. High blood pressure worsens renal function and increases the risk of CVD, therefore, blood pressure control is crucial in patients with DKD [98,99].

The KDIGO recommends a goal of systolic BP of <120 mmHg for CKD patients [66,100]. The ADA recommends a BP goal of <130/80 mmHg for patients with DKD, hypertension, a high CVD risk, and a goal of <140/90 mmHg for patients with DKD, hypertension, and a low CVD risk [66,101]. However, said targets can be individualized [102].

It is recommended that patients with diabetes, hypertension, and albuminuria should be treated with an (renin-angiotensin system) RAS inhibitor, such as an ACEi or ARB, at the highest tolerated dose [66]. Several clinical trials have found that an RAS inhibitor decreased the risk of CKD progression most effectively, compared to a placebo or other antihypertensive drugs [103,104,105]. This is due to their ability to reduce tubular pressure. Since multiple medications are often necessary for a satisfactory BP control, an RAS inhibitor, dihydropyridine calcium channel blockers, and diuretics can be combined to allow patients to reach their individualized BP goals [66].

Non-steroidal mineralocorticoids (ns-MRAs), including esaxerenone and finerenone, which are added to RAS inhibitor therapy, are emerging as new therapies [66]. While esaxerenone has been shown to reduce blood pressure and albuminuria, long-term studies evaluating its effect in patients with DKD are still lacking [106]. The only approved ns-MRA that has slowed the progression of CKD and reduced cardiovascular events in clinical trials is finerenone [107,108,109].

### 4.4. Limitation of Sodium and Protein in the Diet

In patients with CKD, a low-sodium diet and restricted protein intake are an important part of therapy [67,74]. According to the ADA and KDIGO guidelines, low sodium diets of <2.3 g/day and <2 g/day, respectively, aim to maintain adequate blood pressure, reduce cardiovascular risk, and reduce albuminuria [67,74,110], while restricted protein intake reduces the progression of CKD [111,112]. ADA and KDIGO guidelines recommend a protein intake of 0.8 g/kg/day, particularly in patients with stage G3–G5 CKD. In addition, a very low protein diet of 0.3–0.4 g/kg/day, supplemented with essential amino acids or ketoacid analogs, may be used under close supervision in some patients at risk of renal failure [67]. It is important to note that low and very low protein diets cannot be used for metabolically unstable individuals or those in periods of metabolic instability [67].

The randomised trials included in the KDIGO analysis did not show that protein intake <0.8 g/kg/day was associated with significant improvements in renal function or the occurrence of other health outcomes in people with co-existing DM and CKD [113]. There has also been concern that low protein intakes are associated with malnutrition. However, this was not confirmed in the meta-analysis by Hahn et al. [114].

For the National Kidney Foundation Kidney Disease Outcomes Quality Initiative (NKF KDOQI), recommendations for protein intake in patients with DM and CKD are limited to 0.6–0.8 g/kg/day [115]. In patients with renal failure on maintenance dialysis, protein intake may be higher due to concomitant catabolism or malnutrition, and can be as high as 1.0–1.2 g/kg/day [66]. In addition, guidelines recommend the same protein intake in older patients to prevent age-related malnutrition and sarcopenia [116].

### 4.5. Nephrotoxic Agents

An important part of managing DKD is to provide appropriate treatment [117]. Inadequate selection of drugs may result in further renal damage rather than beneficial effects. Moreover, as the GFR declines, the dose of most drugs should be adjusted due to changes in their pharmacokinetics and pharmacodynamics [118]. This ensures that there is no excessive build-up of the drug or its metabolites, which are only removed from the body by the kidneys. In addition, patients with DKD have increased drug sensitivity and, due to their age and comorbidities, reduced tolerance to drug side effects [119].

The 2024 KDIGO guidelines recommend the prudent use of medications and contrast-enhanced studies, weighing their benefits against potential harms before prescribing them [117]. Annually, an estimated 18–20% of patients with advanced CKD are prescribed a nephrotoxic drug, including non-steroidal anti-inflammatory drugs (NSAIDs), antibiotics, proton pump inhibitors (PPIs), or bisphosphonates [120].

Drug-induced injury can affect any structure of the kidney, including the glomeruli, tubules or interstitium. Glomerular damage can result from direct damage to the epithelial cells or endothelium of the filtration barrier, damage to the GBM, damage by the immune response, glomerulonephritis, or chronic glomerulopathy [121,122]. Damage to renal tubular epithelial cells can take the form of acute tubular injury (ATI) or acute tubular necrosis (ATN) [123]. The most common interstitial disease is allergic interstitial nephritis (AIN) [124].

The use of NSAIDs should be limited in patients with CKD as they reduce prostaglandin-dependent renal blood flow, thereby reducing GFR. They may also contribute to AIN or nephrotic syndrome in the form of minimal change disease (MCD) or membranous nephropathy (MN) [118,122,125]. PPIs such as omeprazole, lansoprazole, and pantoprazole can cause tubulointerstitial nephritis. In addition, as with NSAIDs, they may induce AIN, particularly in the elderly, which may contribute to the progression of CKD [126,127]. Lithium and bisphosphonates have been observed to cause both MCD and focal segmental glomerulosclerosis (FSGS) [121,125]. Direct damage to the filtration barrier caused by chemotherapeutic agents such as gemcitabine and mitomycin C or tyrosine kinase inhibitors (TKI), among others, can cause thrombotic microangiopathy [124,128]. Tenofovir and cisplatin may cause renal tubular damage if they accumulate excessively in the renal tubular cells. Tenofovir exacerbates mitochondrial dysfunction and cisplatin forms highly toxic compounds that induce ROS formation, thereby damaging cellular DNA and activating proinflammatory pathways [129]. Among antibiotics, aminoglycosides are the most nephrotoxic. When deposited in proximal tubular cells, they disrupt phospholipid metabolism and cause ATN [130]. In the case of vancomycin, the mechanism of nephrotoxicity is not well understood but is thought to be related to ATN or AIN [131]. AIN has also been reported following the use of sulphonamides, penicillins, cephalosporins, rifampicin, and antivirals such as acyclovir and indinavir [132,133]. There is also increasing evidence of AIN following the use of anticancer drugs including nivolumab and pembrolizumab [124,133]. Sulfonamides may also cause ATN [130]. Crystalluria in the distal convoluted tubule and reversible inhibition of creatinine secretion have also been observed [130]. Similar changes associated with the occurrence of acute crystalline nephropathy have been observed with ampicillin and ciprofloxacin, but also with acyclovir and methotrexate [122,124]. Drug-induced injury to particular renal structures are presented in Table 2.

Given the widespread knowledge of the nephrotoxicity of contrast agents used in imaging studies, it is recommended that the indications for such studies in patients with CKD should be carefully considered [117]. In observational studies, the use of iodinated contrast agents has been associated with the occurrence of acute kidney injury in patients with CKD, hence the current term contrast-associated AKI (CA-AKI) [134,135]. Potential risk factors for CA-AKI include advanced patient age, comorbid heart failure, diabetes mellitus, proteinuria, or the volume of contrast administered [136]. However, treatment or contrast-enhanced studies should not be withheld from potentially critical patients with low GFR [137]. The highest risk of CA-AKI was observed in procedures using intra-arterial rather than intravenous contrast [138]. In addition, AKI occurred more frequently in patients undergoing interventional coronary angiography, which may be related to the larger volume of contrast administered and the additional haemodynamic instability of the patient [136,139]. The incidence of nephrogenic systemic fibrosis (NSF) has been observed with gadolinium used in magnetic resonance imaging [140]. Those at highest risk included patients with AKI, GFR < 30 mL/min/1.73 m^2^, and those on kidney replacement therapy [141].

According to the consensus of radiological societies, intravenous contrast may be administered in patients with AKI or GFR < 60 mL/min/1.73 m^2^, with appropriate precautions [137]. Patients with low GFR may be given low or iso-osmolar contrast at the minimum effective dose for the study. Medications with potential nephrotoxicity should be discontinued in patients 24–48 h before and 48 h after the contrast procedure. Furthermore, dehydration should be avoided in patients undergoing contrast-enhanced procedures [137,142]. The guidelines do not recommend prophylactic peri-contrast haemodialysis, which has been shown to be of no benefit and potentially harmful [143]. In addition, the American College of Radiology group II and III gadolinium-based contrast agents are recommended [117]. The aforementioned recommendations are presented in Table 3.

## 5. Risk Factors and Prevention of Diabetic Kidney Disease

The key element in preventing DKD is the control of risk factors, including strict control of blood glucose levels, BP, treatment of dyslipidemia, and lifestyle changes [144]. Risk factors that lead to the development of CKD in patients with DM can be divided into potentially modifiable and non-modifiable (Table 4) [3].

Undoubtedly, strict glycemia control is very important in order to prevent microvascular [5,145] and macrovascular complications of DM [146]. The basic tool for assessing glycemia control is the determination of HbA1c from blood. Moreover, the HbA1c test has a high predictive value in the context of diabetes complications [147,148]. According to the ADA, the target HbA1c level for most adults should be below 7% to reduce microvascular complications of T1DM and T2DM. In the case of adults who tolerate therapy well with a low risk of hypoglycemia and expected long life, the recommended HbA1c level is below 6.5%. In turn, for older people with a high risk of hypoglycemia episodes, long disease duration, concomitant advanced kidney disease, and expected short life, the recommended HbA1c level is below 8% [149,150]. Another very important aspect in the prevention and treatment of DKD is BP control. Studies show that there is a strong relationship between elevated BP and the constantly deteriorating kidney function in patients diagnosed with DKD [151,152]. The target BP values that should be achieved in people struggling with DKD are still a controversial issue among doctors of many specialties. According to the latest recommendations from the Kidney Disease: Improving Global Outcomes and Kidney Disease Outcomes Quality Initiative guideline committees, the target BP for all patients with CKD should be less than 140/90 mmHg [153]. Studies have been conducted to assess the effect of different BP values in people with DKD. The conclusions from these studies did not prove the benefits of maintaining BP below 130/80 for slowing down the progression of CKD, while at the same time showing an increased rate of adverse events [154]. It should be mentioned that people with DKD have an increased risk of progression of CKD, development of ESRD, cardiovascular events, and death [155,156]. Therefore, appropriate preventive or therapeutic measures are very important to better control risk factors, delay the development of potential complications, and improve the prognosis of patients [157].

## 6. Prognosis

DKD is a very complex disease, initially associated with microalbuminuria or moderately increased urinary albumin excretion (30–300 mg/g creatinine), and, if untreated, it can gradually increase to reach macroalbuminuria within 5–15 years [158]. In addition, it is also associated with a continuous decrease in the GFR. Studies show that, without treatment, the time from the onset of DKD to ESRD is 5 to 7 years [58,159]. Moreover, the main cause of death in these patients was ESRD [58]. The incidence of DKD is constantly increasing, and the diagnosis is often made late, when serious complications are revealed [160]. Taking into account the above aspects, the prognosis of patients is uncertain, therefore prevention, avoidance of risk factors, multidisciplinary treatment aimed at delaying the occurrence of ESRD, and reducing the number of cardiovascular complications are key in care [161,162].

## 7. Conclusions and Future Directions

DKD is one of the most serious chronic complications of diabetes. A number of hemodynamic and metabolic abnormalities contribute to kidney damage in patients with diabetes. Key contributing factors include hyperglycemia, accumulation of AGEs, proteinuria, and lipid overload, leading to structural changes in the kidneys, such as glomerular hypertrophy and basement membrane thickening. The damage affects both glomerular and tubular cells, promoting renal fibrosis, inflammation, and oxidative stress. DKD is characterized by decreased renal function, proteinuria, and diabetic retinopathy, with albuminuria being a key diagnostic marker. In diagnostics, we assess albuminuria using ACR tests and calculate eGFR, and assign one of four histological classes based on severity. Patients diagnosed with DKD should be treated with comprehensive care by a team of multiple specialists. Lifestyle modification with changes such as an optimized diet and exercise, smoking cessation, and weight management should be the foundation of the treatment. First-line therapy for an optimized glycemic control consists of metformin and an SGLT2 inhibitor, as this allows for the best health benefits. If needed, other additional antidiabetic drugs can be added to the base-line therapy, and the preferred medication is a GLP-1 receptor agonist. Hypertension and dyslipidemia are common comorbidities in patients with DKD. Statin therapy is recommended for optimal lipid management and as a form of CVD prevention. High blood pressure should be treated with an RAS inhibitor, such as an ACEi or ARB, at the highest tolerated dose. Risk factors for DKD are divided into non-modifiable (e.g., age, genetics) and modifiable (e.g., poor glycemic control, hypertension). Prevention of DKD requires these risk factors to be controlled, in particular blood glucose levels and blood pressure to delay the progression of kidney damage.

This review has limitations. There were no specific selection criteria or research methodology used to identify sources and references for this review. Due to the extent of the topic, the focus was on the pathophysiology and diagnosis of diabetic kidney disease, key recommendations for patients, and prevention of the onset of the disease. In addition, the mechanisms of renal damage of the most commonly used drugs, not all of which have been recognised as nephrotoxic, are listed.

The way forward is to take advantage of the knowledge of the pathophysiological mechanisms that are important in diabetic kidney disease to develop treatments that act directly on these mechanisms. Furthermore, it is important to seek treatments that can reverse, as far as possible, the renal changes that have occurred. It is also important to provide patients with comprehensive care and to review the medications they are taking that may be contributing to the deterioration of kidney function.

## Figures and Tables

**Table 1:** Classification of urinary albumin concentration according to the 2012 KDIGO guidelines [60].

Measure	Normal or Mildly Increased (A1)	Moderate Increased (A2)	Severely Increased (A3)
Albumin excretion rate (mg per 24 h)	<30	30-300	>300
Albumin-to-creatinine ratio (mg/g)	<30	30-300	>300
Albumin-to-creatinine ratio (mg/mmol)	<3	3-30	>30

**Table 2:** Summary of the location of drug-induced kidney damage [118,121,122,124,125,126,127,128,129,130,131,132,133].

The Location of Kidney Damage	Medicines
Glomerular	NSAIDs, lithium, bisphosphonates, chemotherapeutics—gemcitabine, mitomycin C, antiangiogenesis drugs—tyrosine kinase inhibitors targeting VEGFR
Tubular	Proton pump inhibitors—omeprazole, lansoprazole, pantoprazole; antivirals—tenofovir, acyclovir; antimicrobials—aminoglycosides, vancomycin, sulfonamides, ampicillin, ciprofloxacin; chemotherapeutics—cisplatin; methotrexate
Interstitial	NSAIDs, proton pump inhibitors—omeprazole, lansoprazole, pantoprazole; antimicrobials—vancomycin, sulphonamides, penicillins, cephalosporins, rimfapicin; antivirals—acyclovir, indinavir, checkpoint inhibitors—nivolumab, pembrolizumab

**Table 3:** Summary of guidelines on the use of contrast agents in patients with CKD [117,137,143,144].

Recommendations from the Consensus of Radiological Societies
The use of low or iso-osmolar contrast agents
The use of group II and III gadolinium-based contrast agents
The use of minimum effective dose of contrast agent for the diagnostic study
Discontinuation of potentially nephrotoxic medications 24–48 h before and 48h after contrast procedures
Avoidance of dehydration in patients undergoing contrast-enhanced procedures
Prophylactic peri-contrast haemodialysis should not be performed

**Table 4:** Modifiable and non-modifiable risk factors for diabetic kidney disease [3].

Not Modifiable	Modifiable
Advanced age Early age at onset of diabetes Long-term duration of diabetes Family history of diabetic kidney disease, type 2 diabetes, non-diabetic kidney disease, hypertension, or insulin resistance Intrauterine growth retardation Maternal gestational diabetes or developmental glucose exposure Genetic factors Ethnicity	Poor glycemic control Hypertension Dyslipidemia Unfavorable socioeconomic position Sedentary lifestyle or low intensity of physical activity Obesity Smoking Insulin resistance or metabolic syndrome Recurrent or chronic infections Episodes of acute kidney injury Use of oral contraceptives Hyperuricemia Vitamin D deficiency

## Data Availability

The data used in this article were sourced from materials mentioned in the References section.
